# DNA microarray integromics analysis platform

**DOI:** 10.1186/s13040-015-0052-6

**Published:** 2015-06-25

**Authors:** Tomasz Waller, Tomasz Gubała, Krzysztof Sarapata, Monika Piwowar, Wiktor Jurkowski

**Affiliations:** 1 Institute of Computer Science, Division of Biomedical Computer Systems, University of Silesia, Katowice, Poland; 2 Academic Computer Centre CYFRONET, AGH University of Science and Technology, Kraków, Poland; 3 Molecular Biology and Clinical Genetics Laboratory, Department of Medicine, Jagiellonian University, Kraków, Poland; 4 Department of Bioinformatics and Telemedicine, Medical College, Jagiellonian University, Kraków, Poland; 5 The Genome Analysis Centre, Norwich, UK

## Abstract

**Background:**

The study of interactions between molecules belonging to different biochemical families (such as lipids and nucleic acids) requires specialized data analysis methods. This article describes the DNA Microarray Integromics Analysis Platform, a unique web application that focuses on computational integration and analysis of “multi-omics” data. Our tool supports a range of complex analyses, including – among others – low- and high-level analyses of DNA microarray data, integrated analysis of transcriptomics and lipidomics data and the ability to infer miRNA-mRNA interactions.

**Results:**

We demonstrate the characteristics and benefits of the DNA Microarray Integromics Analysis Platform using two different test cases. The first test case involves the analysis of the nutrimouse dataset, which contains measurements of the expression of genes involved in nutritional problems and the concentrations of hepatic fatty acids. The second test case involves the analysis of miRNA-mRNA interactions in polysaccharide-stimulated human dermal fibroblasts infected with porcine endogenous retroviruses.

**Conclusions:**

The DNA Microarray Integromics Analysis Platform is a web-based graphical user interface for “multi-omics” data management and analysis. Its intuitive nature and wide range of available workflows make it an effective tool for molecular biology research. The platform is hosted at https://lifescience.plgrid.pl/.

## Background

Transcriptomics, lipidomics and other molecular techniques produce enormous volumes of data that must be stored, analysed and interpreted using various methods. The integration of “multi-omics” data displays the potential to fully expose the molecular mechanisms occurring in an organism. A holistic approach to biomedical research may help identify new biomarkers for disease diagnostics and improve the sensitivity and specificity of the existing biomarkers [1]. The inclusion of temporal and spatial parameters enables mathematical modelling (often referred to as “systems biology”), which may produce new insights into the mechanisms of pathogenesis and support the development of novel therapies [1].

Comprehensively cataloguing the interactions between genes, lipids and other biological molecules is a highly complex task. Although the human genome contains just over 25,000 genes, the human metabolome – given the multitude of post-translational modifications – is composed of millions of different molecules. Genomes, transcriptomes, and proteomes are fundamental to the functional integrity of the organism. These metabolites reflect the important functions of gene and protein regulation; thus, genomics, transcriptomics and proteomics may provide vital information regarding the biological status of the system. Modelling and studying the interactions between molecules belonging to different biochemical families (proteins, nucleic acids, lipids, and carbohydrates) requires large-scale computing power and specialized data analysis methods.

DNA microarrays represent a high-throughput measurement technology that is widely used in biological research, especially gene expression experiments. This technology revolutionized biological research by enabling the discovery of a large set of genes whose expression levels reflect a given cell type, treatment, disease or developmental stage [2]. In the first quarter of 2014 alone more than 1600 experiments based on DNA microarrays were uploaded to the functional genomics experiment database operated by the European Bioinformatics Institute (termed ArrayExpress [3]). Integrating DNA microarray data with datasets from external sources may improve the identification of significant biomarkers [4].

The analysis of environmental and hereditary factors begins with a snapshot of the transcriptome using a set of probes. The lipidome and the microRNAome can guide the exploration of gene-level mechanisms. Integrating the transcriptomics and lipidomics data with microRNA (miRNA)-mRNA interaction data may reveal new information about the underlying cellular processes that cannot be directly derived from any of the individual datasets.

At the heart of mRNA-miRNA interaction analysis is the correct identification of miRNA-corresponding targets. This identification is facilitated by various computational algorithms and laboratory experiments. However, the gene regulation processes involving miRNA are poorly understood, resulting in low specificity and poor accuracy of the targets identified using the available prediction methods [5]. To improve target site recognition we should exploit additional information: expression level measurements of both miRNA and mRNA, evaluation of targets obtained using other prediction methods, sequence-based information, contextual information, phylogenetics and experimentally validated databases. To integrate these sources of information scientists use regression [6, 7] and correlation methods [8–10].

Although several web-based DNA microarray analysis platforms have already been developed [11–13], most do not support integration of “multi-omics” data. In contrast, the DNA Microarray Integromics Analysis Platform permits integrated analysis of transcriptomics and lipidomics data, along with analysis of miRNA-mRNA interactions.

## Implementation

**Fig. 1:**
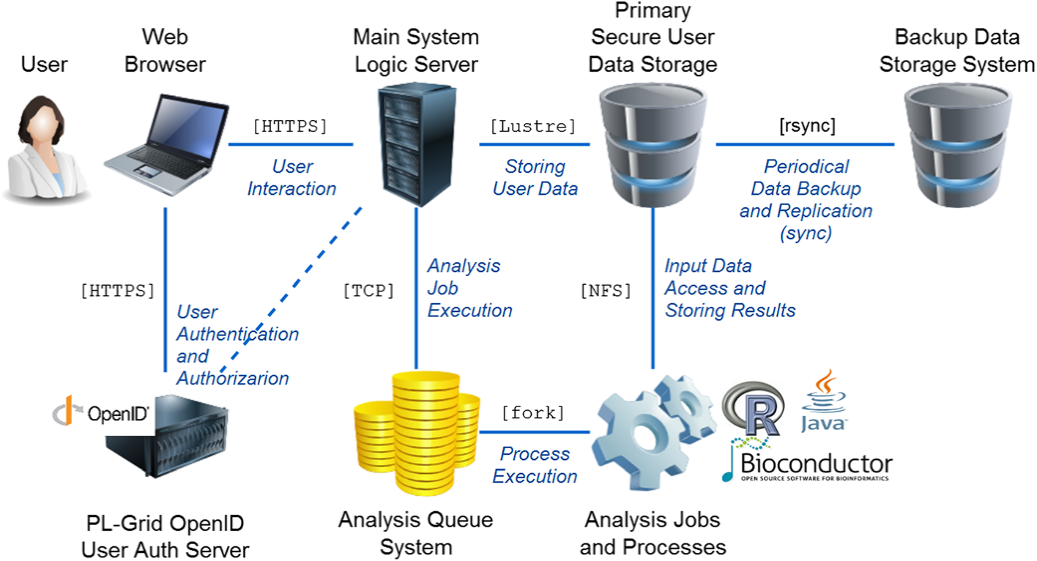
This diagram displays the setup of the DNA microarray analysis platform. The main web server performs third-party authentication using PL-Grid OpenID, and subsequently serves authorized content. Large-scale computations are performed asynchronously in a job queue and all user data is backed up to secure secondary storage for additional fault tolerance

The platform (see Fig. 1) is provided to users in the form of a web application. To ensure a powerful, stable and sustainable foundation for the presented system, the PL-Grid Infrastructure [14] was selected as the underlying hardware layer. Any user data uploaded to the platform is stored in a secure disk array based on Lustre technology, which is important from the point of view of efficient computation (i.e., rapid parsing and analysis of microarray data). All user data is periodically backed up using an efficient synchronization protocol (rsync) to a redundant storage system. This additional system is located on a physically separate rack server at an offsite location, providing further fault tolerance and user data safety.

The most important function of the system is to provide a rich set of available algorithms for gene expression analysis. To sustain efficient user interfaces in the web application portion (the *frontend*), the platform offloads all heavy-duty analytical tasks to background processes. All user analysis tasks are managed by a standalone queuing system that the main platform uses to schedule new analysis jobs, monitor the execution status of existing jobs and notify the user when a job is complete. Such an asynchronous mode of operation allows researchers to freely use the system while waiting for scheduled tasks to complete. The queue functions in a FIFO mode for all user jobs such that no user is privileged and all users share common computational resources according to a *fair share* policy. Currently, our platform provides a set of analyses based on R Bioconductor packages [15] and custom Java solutions. The web application portion of the system was developed using the Ruby on Rails framework. The platform is an open source project [16].

The DNA Microarray Integromics Analysis Platform supports a wide range of analyses, including primary raw data processing and detection of differential expression, as well as more advanced techniques, including data mining procedures explaining gene expression-phenotype relationships (clustering, multidimensional scaling, and construction of predictive models). The prime focus of the Analysis Platform is the integration of various ‘omics’ data – accordingly, two specific features (integration of lipidomics and transcriptomics data; integration of miRNA and mRNA data) are described in more detail in the following sections.

### Low-level analysis of DNA microarray data

Low-level analysis is the first step in the analysis of DNA microarray data. The DNA Microarray Integromics Analysis Platform supports different types of Agilent (e.g., SurePrint G3 4 × 44 K or SurePrint G3 8 × 60 K) and Affymetrix (e.g., Mouse Gene 1.0 ST; Human Gene 1.0 ST; or U133A 2.0) one-channel DNA microarray data. The platform is able to perform background adjustment, normalization and summarization of raw DNA microarray data based on widely accepted methods and algorithms. There are three options for background adjustment (no background correction, RMA [17], or GCRMA [18]) and three options for data normalization (quantile [19], scaling and variance stabilization [46]). Once the preprocessing step is complete, any differences between the microarrays are confirmed to be due to differential expression rather than printing, hybridization, or scanning artefacts.

### Differential analysis of the gene expression and metabolite levels

To calculate differences in the transcript levels, the platform supplies two methods: Significance Analysis of Microarray (SAM) and *T*-test statistical analysis. Significant gene selection via SAM [21] calculates the relative differences in the transcript levels based on statistical analysis of data permutations.

Both methods can be applied separately to miRNA and mRNA expression data. The latter is also used to determine the differential levels of small-molecule concentrations as measured by mass spectrometry experiments.

In addition to the aforementioned approaches which directly depend on comparisons of individual transcript levels, the platform also enables the use of functional priors in the form of Gene Set Enrichment Analysis (GSEA), which is commonly used to test for statistically significant overrepresentation of particular groups of genes in the context of the Gene Ontology Annotation Database [24].

### Data clustering

Data structure can be investigated by performing cluster analysis which categorizes unsupervised data into classes of relative similarity using various samples or experiments (represented as a dendrogram) [20]. The platform supports clustering based on samples or transcripts. The user may select different methods of distance measurement and agglomeration. Cluster analysis can be used to identify novel sample clusters and their associated transcripts, and to evaluate data quality by verifying that replicate samples under similar conditions are clustered together.

The platform also includes Principal Component Analysis (PCA) to reduce the dimensionality of the data by extracting the variables (coordinates) displaying the highest variance, which are assumed to account for the variability in the analysed dataset [23].

### Phenotype classification

Conditional analysis of gene expression based on artificial neural networks (ANNs) is capable of creating and training a neural model (a perceptron) and subsequently using it to distinguish between healthy and abnormal tissue based on gene expression profile data [22]. This ANN analysis supports the simultaneous training of thousands of network architectures, computing predictions from each network architecture to pinpoint the single best network. Trained ANNs have been successfully applied to the analysis of various diseases, including the classification of cardiovascular disease and the prediction of bacteria-antibiotic interactions and colorectal cancer patient survival [22].

### Analysis of biological pathways

Biological pathways can be currently accessed using the Wikipathways [25], KEGG [51] and Reactome [52] web services as an optional step following determination of the differentially expressed genes. The platform scans the gene expression datasets and the pathway repositories to identify the genes that participate in a genetic pathway. The genes in the specified pathway are graphically represented as a color-coded diagram. The discovered pathways are ranked by the percentage of genes affected.

### Analysis of transcriptomics and lipidomics data

Although multivariate statistical techniques are the method of choice for studying complex data relationships, classical methods such as canonical correlation analysis (CCA) or partial least squares regression (PLS) [26] are not practical due to the specific structure of the data obtained from high-throughput platforms. For example, gene expression data typically include tens of thousands of variables measured from a small number of observations (samples taken under certain conditions). To cope with this suboptimal data structure comprising many variables and few observations, the standard methods can be modified to restructure data either by performing feature selection or introducing artificial variables. Sparse PLS (sPLS) reduces the number of variables incorporated into the model [27, 28] and can be applied to both regression and canonical correlation frameworks. Regularized canonical correlation analysis (rCCA) increases the number of pseudo-observations in the regularization procedure of covariance matrices [29, 30]. Finally, principal component regression (PCR) [31] represents the class of multidimensional scaling techniques. Despite difficulties in estimating statistical significance, appropriate application of these methods, including correctly assuming the correlations between variables, provides an approximate interpretation of heterogeneous data (e.g., metabolites and genes), which can be helpful for biological data analysis and may yield clinically relevant results [32–34].

All statistical techniques included in the presented platform are implemented in R and include classical CCA and rCCA (YACCA [35] and FRCCA [36]) and PLS [37], as well as specialized packages dedicated to lipidomics data (mixOmics [38]).

**Fig. 2:**
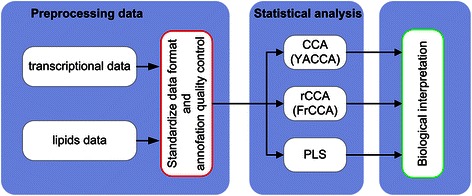
Integrated transcriptomics and lipidomics data analysis workflow

Integration of data obtained from two independent platforms is based on three statistical methods: classical canonical analysis (CCA; YACCA package in R), regularized canonical analysis (rCCA; FRCCA package in R) and PLS (see Fig. 2).

### Analysis of miRNA-mRNA target site recognition

The interactions between miRNAs and mRNAs play an important role in the process of gene regulation. RNA interference (RNAi) depends on appropriately recognizing specific targets of the miRNA molecules that are integrated into an RNA-induced silencing complex (RISC) [39]. RISC uses miRNA as a template for recognizing complementary transcripts. However, in metazoans this complementarity is imperfect. Generally, a miRNA-mRNA duplex occurs in the 3′UTR region, and complementarity applies only to the first 7–8 nucleotides of the miRNA, also referred to as the seed. Such short complementarity is a fundamental problem for correct recognition of the target site, as its specificity is very low. As a consequence, it is possible for any miRNA-mRNA pair to interfere. Additionally, mismatches may occur in the seed region of the miRNA during the hybridization process.

We use TargetScan [40] to predict miRNA targets. The method considers the 3′UTR regions of the transcriptome and calculates context score [41] from the type of seed match (8mer, 7mer-m8, and 7mer-1A), 3′ pairing contribution (miRNA-target complementarity outside the seed region); preferentially conserved targeting (PCT) estimated as described in [42] and [43]; and multiple additional parameters.

Effective expression differentiation and miRNA/mRNA interaction detection require large sets of samples representing multiple cell types or conditions. As sufficiently large sample sets are unfortunately not always available. The platform supports the inference of miRNA-mRNA interactions using two complementary Bioconductor methods, Roleswitch [44] and TargetScore [45], which account for these limitations.

Roleswitch is able to infer probabilities of miRNA-mRNA interactions from a single sample. The Probabilities of MiRNA-mRNA Interaction Signature (ProMISe) are calculated from two paired expression vectors, M miRNA and N mRNA, and the N x M seed match matrix containing the number of target sites for each miRNA-mRNA pair. Notably, Roleswitch assumes that the total transcribed mRNA levels are higher than the observed (equilibrium) mRNAs levels. An entire family of versatile preliminary seed matrices (in which the source data is obtained from the TargetScan database) has been prepared and is provided to platform users. The pre-calculated matrices differ with respect to the following:

- conservation of miRNA: broadly conserved = conserved across most vertebrates, typically up to zebrafish; conserved = conserved across most mammals, but typically not beyond placental mammals; or poorly conserved = all others; and
- the threshold of the total context site, defined as the sum of context scores for the most favourable (most negative) miRNA in this family. For highly conserved miRNA families the total context site is complementary to preferential conservation of the sites (aggregate PCT).

The user may select targets of interest, including the expected level of conservation.

The TargetScore approach assumes input data from the experimental transfection procedure. Transfection relies on the introduction of miRNA molecules to cells containing the reagent. By determining the expression levels of transcripts prior to and following transfection one can infer the targets of the given miRNA molecule. In this model, paired microarrays of mRNA and miRNA are generated from the same experiment to measure the cellular response to an environmental change (e.g., treatment with endotoxin lipopolysaccharides (LPS)). When multiple miRNA samples are examined a list of miRNA molecules is used as the input, as in the case of a transfection experiment, into a corresponding mRNA expression matrix in the log(FC) form. Then, the miRNA-mRNA interactions are analysed using the TargetScore package and the appropriate sequence-based information (including the context score and PCT) for each selected miRNA sample.

TargetScore infers the posterior distributions of miRNA targets by probabilistically modelling the likelihood of a fold-increase in mRNA expression and sequence-based scores. The variational Bayesian Gaussian mixture model (VB-GMM) applies these logarithmic fold-change and sequence scores to determine the posterior latent variables (i.e., the miRNA targets). The sequence-based information, context score and PCT are obtained from the previously mentioned TargetScan database. The author of TargetScorehave demonstrated that target prediction can be improved by simultaneously using expression fold-change and sequence-based information. The advantages of TargetScore include improved quality of target recognition via the use of sequence-based information and its deployment as an unsupervised method operating on the entire gene dataset to more closely model the overall interaction probability.

Using the presented DNA microarray analysis it is possible to investigate miRNA-mRNA interactions based on the following: 1) a single sample; 2) differential expression; 3) differential expression and sequence-based scores; or 4) a combination of the above approaches.

Given a set of paired raw miRNA and mRNA microarray expression data from each sample, the investigation workflow follows a typical normalization and expression-calling procedure, as well as steps specific to integrated miRNA and mRNA analysis.

Predictions are summarized by listing the genes/transcripts ranked by the mRNA samples displaying the maximum probabilities of interaction with any miRNAs, the miRNA samples displaying the maximum probabilities of interaction with any transcripts and the maximum probabilities of interaction between specific miRNA-mRNA pairs.

The user is able to operate not only at the gene level but also at the transcript level. Most R/Bioconductor microarray workflows function only at the gene level, by associating a single gene with a set of probes and calculating the average expression for multiple transcripts of the same gene. However, it is occasionally more effective to focus on the transcripts, such as when investigating gene isoforms or alternative splicing. When the user selects transcript-level analysis, the platform links the related transcripts to the same gene in the analysis results. In the inverse case, in which a single transcript is related to multiple genes, the system presents the user with a list of such transcript-gene pairs.

### Data management

The DNA Microarray Integromics Analysis Platform supports simple and efficient data management in a variety of modalities.

**Fig. 3:**
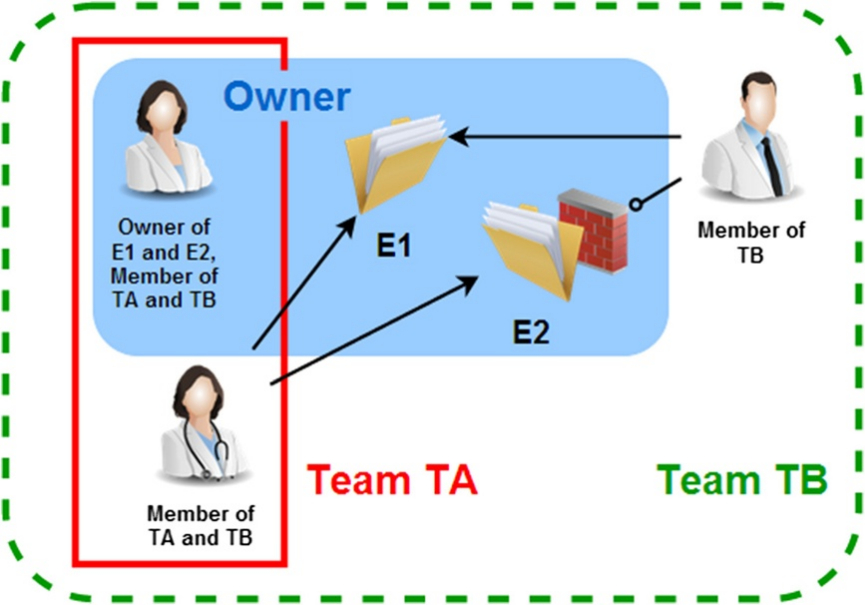
Sharing mechanism. Based on the dynamic definition of a team, the owner of an experiment (a set of data, analyses and results) is able to share it with his/her collaborators. Each user may join any number of teams. Within each team, membership is supervised by the team manager (often the PI of a research project). Here, the owner shares experiment E1 with team TB and shares experiment E2 with team TA, resulting in the access rights presented in the diagram

No researcher works alone. To deliver experiment-sharing capabilities (an “experiment” is defined as a set of connected raw data variables, analyses and their results), the platform implements a team-based sharing mechanism. A user is able to share an experiment with any team he/she belongs to (see Fig. 3).

To accommodate both users who are in the middle of their experimentation and data analysis process and those who have already published their results, the platform provides two important mechanisms, both of which are based on the Minimum Information About a Microarray Experiment (MIAME) standard [47]. First, the system enables its users to import preprocessed datasets by uploading a matrix of normalized transcript expression data containing rows representing individual transcripts and columns representing samples (individual microarrays). Using an SDRF file prepared according to the MIAME regulations, the user uploads both the preprocessed data and the experimental metadata to the system. Once the experiment has been appropriately registered, it is possible to conduct high-level analyses using the imported data.

When a user has already published his/her findings in a journal and uploaded the data to the EBI ArrayExpress [3] database, the platform facilitates importing the entire experiment directly from this database. Experimental data is downloaded in the background upon request – the users are not required to handle any files themselves. If the experimental record in the ArrayExpress database contains all of the data necessary for the system to process it and the microarray files are of a type recognized by the platform, the experiment is downloaded, and the platform notifies the user when the download process is complete. This feature is also useful for educational purposes, so that a teacher and students do not need to supply their own data (which could be cumbersome in the case of large microarray experiments), but rather can simply select an interesting publicly accessible ArrayExpress experiment and rely on the platform to download all of the necessary data for them. The system uses a high-throughput backbone network that is a component of the PL-Grid Infrastructure, which ensures rapid transfer between European laboratories and the disk array of the system.

The platform also helps export microarray experiments to the ArrayExpress database by providing a facility to easily generate the required MAGE-TAB files (specifically, Investigation Description Format (IDF) files and Sample and Data Relationship Format (SDRF) files) according to the required MIAME data and formatting regulations. These files are useful when registering one’s own microarray experiments in the ArrayExpress database.

## Results

### Case study 1: functional links between gene products and metabolites

Publicly available lipidomics and transcriptomics data from a murine nutrigenomics study [48] were used to demonstrate the performance of the proposed analytical protocol using two independent data sets. The data consist of the expression levels of 120 genes (X) and the concentrations of 21 fatty acids (Y), both spanning a total of 40 observations of several genotypes and diets.

**Fig. 4:**
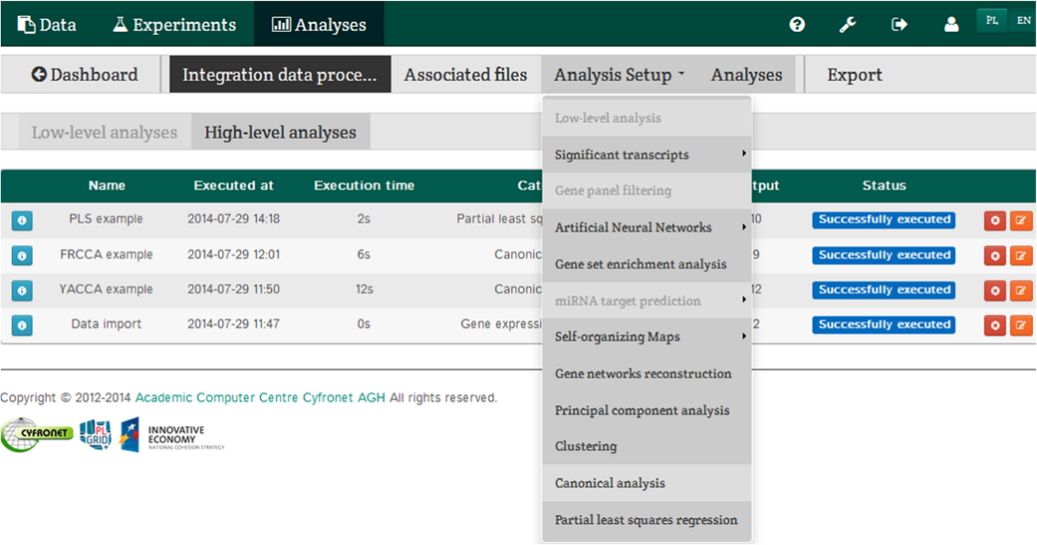
The “Analysis Setup” panel enables the selection of analytical methods, such as canonical analysis (CCA or rCCA) or PLS

Following data import, analytical procedures were executed using the Integromics platform (Fig. 4). Depending on the number of observations compared to the number of variables (dependent and independent), appropriate methods of canonical correlation (i.e., classical canonical correlation, such as CCA or rCCA) may be applied.

CCA is appropriate for cases involving a relatively large number of observations and a low number of correlated variables (dependent and independent) in the two datasets. rCCA is a modification of the classical canonical correlation method that permits analysis of a small number of observations and a large number of variables in both sets. PLS is not restricted by the number of observations or highly correlated variables.

#### Case study using YACCA (canonical analysis) and FRCCA (regulated canonical analysis) packages in R.

The canonical analysis procedure (CCA) generates specific data corresponding to the analysed datasets. This case study focused on the most basic parameters of canonical analysis and their interpretation. Conducting CCA initially requires the removal of correlated predictors (independent variables, or the transcriptomics data (X)) and responses (dependent variables, or the lipidomics data (Y)) as highly correlated variables may significantly skew the results. The correlation threshold is set arbitrarily and depends on the type of data. In the life sciences domain, the correlation coefficient is considered to be greater than 0.7.

**Fig. 5:**
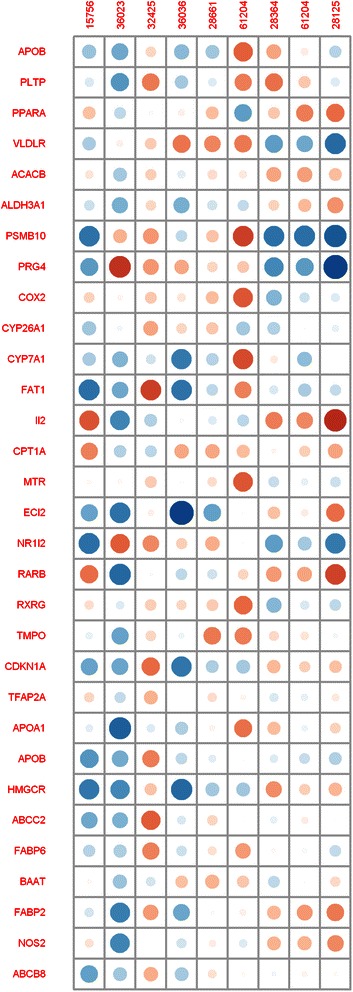
Map of the Pearson correlations between two datasets: gene transcripts (X) and fatty acids (Y). The radius of the circle indicate the strength of correlations. Negative correlations are marked in blue while positive correlations are marked in red

Once highly correlated data are removed from X and Y, the correlation between the dependent and independent variables can be calculated. The Integromics platform produces a correlation map for X and Y, identifying the variables that display strong correlations (Fig. 5).

The appropriately prepared data sets were analysed using the canonical correlation procedure which identifies linear combinations of variables in the analysed sets such that the correlations between them are as high as possible. The results of the CCA procedure are accessible as text (numerical data) or PNG (figures) files several seconds after the analysis is complete.

Detailed information about the CCA results, such as the canonical correlations, the X coefficients, the Y coefficients, the structural correlations (loadings) for variables X and Y, and the aggregate redundancy coefficients (total variance explained) are accessible in the cca.fit-YACCA.txt file. Statistics that describe datasets X and Y are saved in the summaryX-YACCA.txt and summaryY-YACCA.txt files respectively. The significance of the calculated canonical correlation is performed using the F-test (Rao’s F approximation), and these results are provided in the F_test_CCA.txt file.

The results obtained from the murine nutrigenomics datasets reveal high canonical correlations (CV1:1, CV2:0.997, CV3:0.995). These canonical correlations were found to be statistically significant at a significance level of 0.05. The calculated values of aggregate redundancy were X | Y: 0.42 and Y | X: 0.89, indicating that Y explains X in 42 % of cases and that X explains Y in 89 % of cases.

**Fig. 6:**
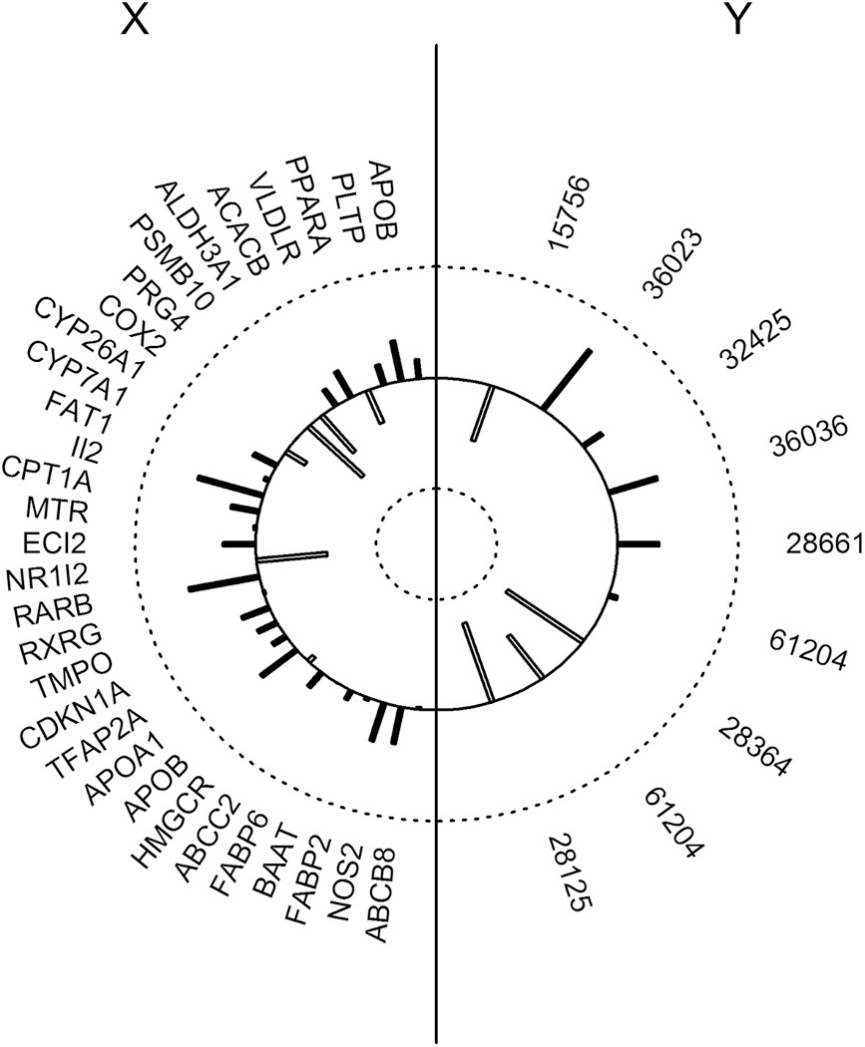
Graphical representation of the canonical loadings of the X (gene transcript) and Y (fatty acids) datasets for the first canonical variates (CV1)

The platform supplies graphical files which present the results of the analyses. For example, a diagram of the impact loadings in both sets is presented in Fig. 6.

High canonical loading of a particular variable indicates the significance of that variable in the interpretation of the canonical variate. In this case, among the gene transcripts, the greatest impact on this model was obtained for genes PRG4 (−0.637), Il2 (0.56), NR1I2 (−0.58), and RARB (0.59) and for fatty acids 28364 (−0.78), 28125 (−0.75), and 36023 (0.66). These results are reflected by the heights of the bars in the graph.

As mentioned above, CCA should be performed for a large number of observations. If the number of observations is lower than the number of transcriptomics or lipidomics variables, classical canonical analysis is not appropriate (the number of observations should be 10 times the number of variables). In the life sciences domain the obtained data often displays a structure in which the number of observations (samples) is small while the number of variables (e.g., transcripts of genes) is large. In such situations, a modified classical canonical analysis method (rCCA) should be used.

For the analysed dataset, CCA can be used when the correlation threshold is at least 0.7 for the transcriptomics andlipidomics data. When *r* = 0.8, CCA is not performed due to the insufficient number of observations compared to the number of X and Y variables. In such casesrCCA can be performed instead. The results of the rCCA calculation are available in a similar form to the CCA results.

**Fig. 7:**
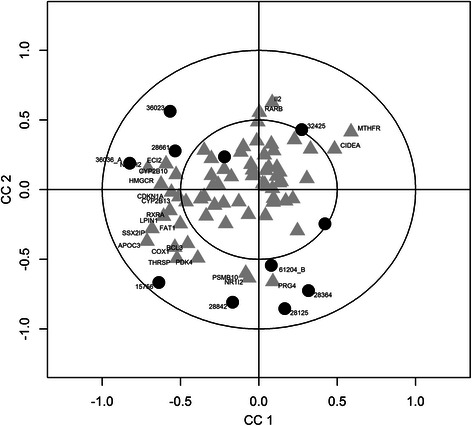
Canonical variable representation

The canonical correlation and the canonical weights and loadings for X and Y are accessible in the my_res-FRCCA.txt file. Descriptive statistics are provided in the summaryX-FRCCA.txt and summaryY_FRCCA.txt files. In FRCCA the results do not include information regarding the significance calculations. The values of the canonical correlations for the first three sets of canonical variables are as follows: CV1: 0.99; CV2: 0.98; and CV3: 0.98. The graphical representation depicts the variables as points on a two-dimensional plane in which the axes represent the canonical loadings, as shown in Fig. 7.

#### PLS procedure

For the integration of partially correlated transcriptomics and lipidomics data the PLS procedure was used. The first step of the PLS procedure involves constructing a predictive model. For this purpose the entire dataset was incorporated into the PLS model.

**Fig. 8:**
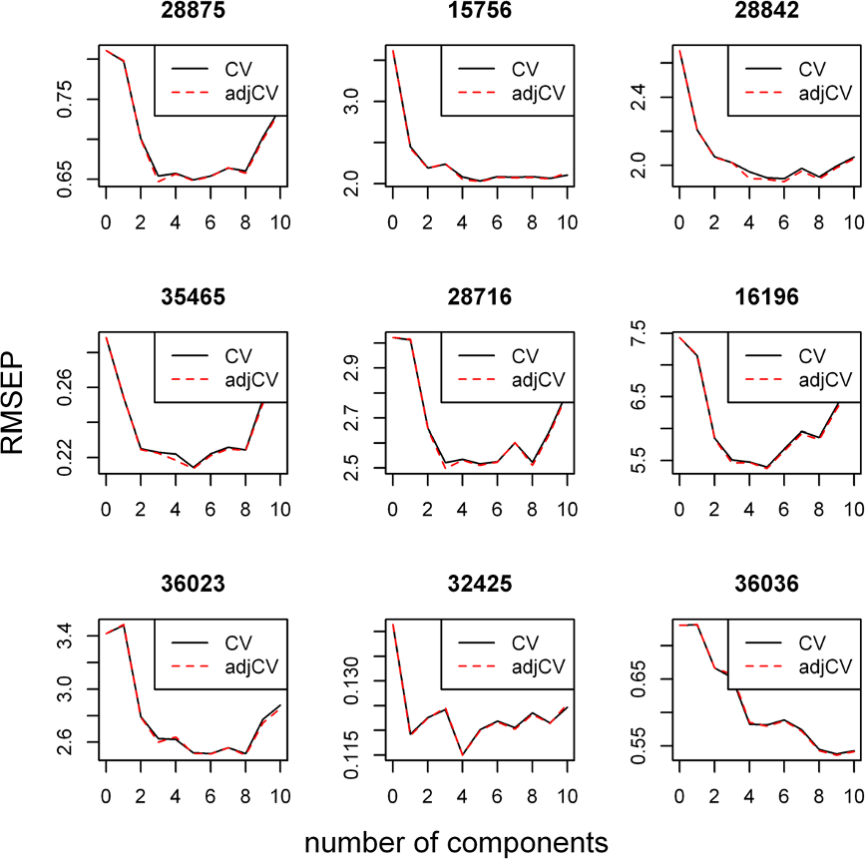
Cross-validated RMSEP curves for the murine nutrigenomics dataset (results for the first 9 lipidomics variables). “CV” is the cross-validation estimate while“adjCV” (for RMSEP) is the bias-corrected cross-validation estimate. They can only be calculated if the model has been cross-validated

To demonstrate the prediction values, a subset of murine nutrigenomics data were used (50 transcriptomics and 10 lipidomics variables). The PLS model was fit to the 10 latent variables, followed by cross-validation. The root mean squared error of prediction (RMSEP) parameters were calculated for the specific lipidomics variables (fit method: kernelPLS; number of components considered: 10). This step yields information about the quality of the fit of the model to particular components. In the analysed dataset, some variables were classified as poor (RMSEP too high), such as ChEBI 16196, in which RMSEP was >4.5 for ncomp = 8 and even higher for ncomp > 8 (Fig. 8). Other variables remained good, such as ChEBI 15756, exhibiting the lowest RMSEP in 5 components.

**Fig. 9:**
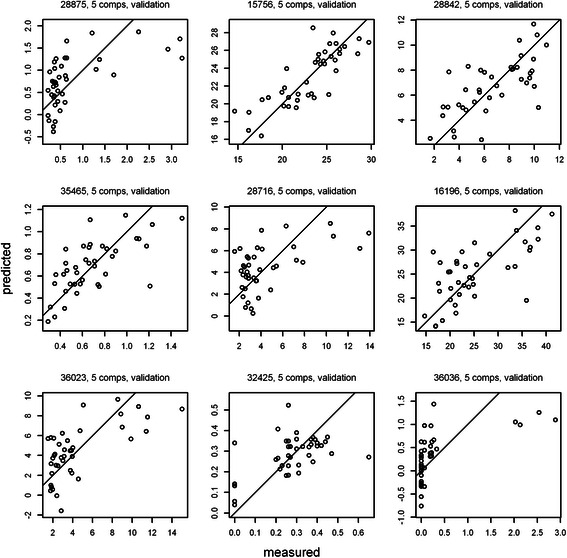
Cross-validated predictions for the murine nutrigenomics dataset (results for the first 9 lipidomics variables)

To confirm the quality of the variables included in this model, cross-validated predictions using 5 components (the components for which most of the variables displayed the lowest RMSEP) were visually compared to the measured values (Fig. 9).

**Fig. 10:**
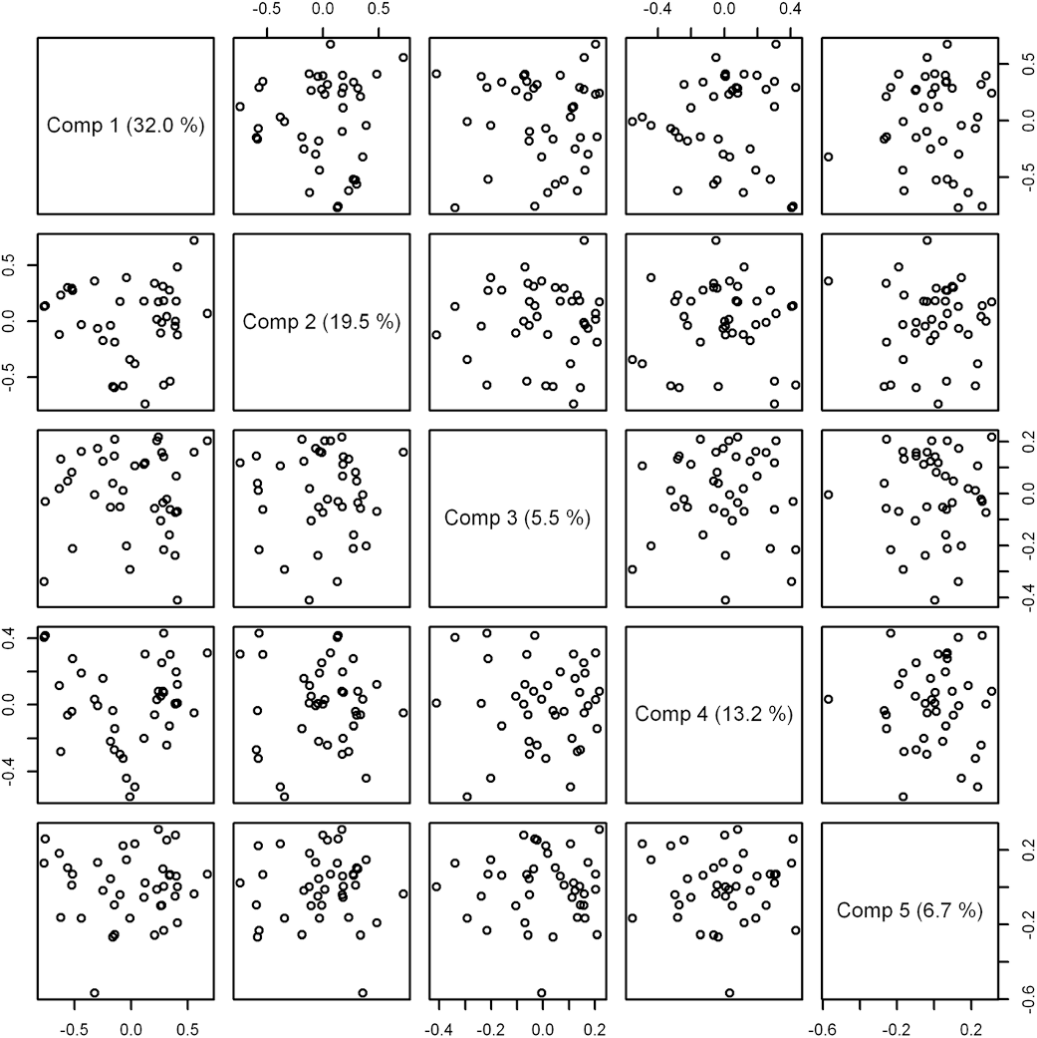
Score plot for the murine nutrigenomics dataset

The charts of some of the variables exhibited poor alignment to the corresponding target profiles – e.g., fatty acid ChEBI ID 36036 (Fig. 9). This result suggests that the X variables did not appropriately describe the given lipidomics variable. Another graphical representation of the results of the PLS procedure consists of a pairwise plot of score values. Such plots help reveal patterns, groups and outliers. In our example (5 components plotted), there was no clear indication of any grouping or outliers (Fig. 10).

**Fig. 11:**
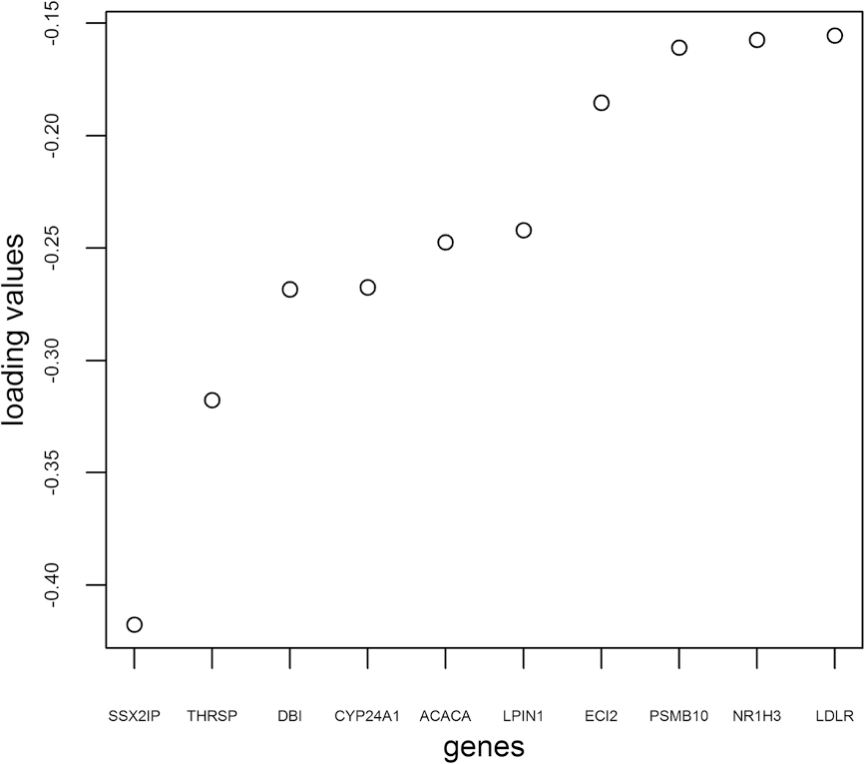
Top 10 genes ranked by their loading values

The explained variances for each component in this example were as follows: Comp1 = 32 %, Comp2 = 19.5 %, Comp3 = 5.5 %, Comp4 = 13.2 %, Comp5 = 6.7 %, Comp6 = 4.2 %, Comp7 = 3.6 %, Comp8 = 2.1 %, Comp9 = 1.4 %, and Comp10 = 1.1 %. The canonical loadings are crucial for the interpretation step as higher loading values indicate greater contribution of the given variable to the model. Figure 11 presents the top 10 genes, ranked according to their loading values.

### Case study 2: discovery of significant miRNA interactions

The other use case concerns the Normal Human Dermal Fibroblasts (NHDF) experiment [49]. This study compared the differential expression of the entire transcriptome, including miRNAs, between different cell lines (12 samples): a control group of NHDF cells (NHDF), NHDF cells treated with LPS (NHDF LPS), NHDF cells infected with porcine endogenous retroviruses (PERVs) (NHDF PK15) and NHDF PERV-infected cells treated with LPS (NHDF LPS PK15). For each sample, the paired expression levels of both mRNA and miRNA were measured using AffymetrixGeneChip miRNA 2.0 and HGU133A2 microarrays. This study measured the changes in the expression levels of both mRNAs and miRNAs between specific groups and, when changes were found, attempted to establish which RNAi process regulated their expression in PERV-infected cells either with or without LPS treatment.

Preliminary miRNA microarray data preparation was performed using the Affymetrix miRNA QCTool [50]. The procedure included background detection, background adjustment (BC-CG Adjust), quantile normalization, addition of small constants (CVStab), and summarization using a median polishing approach. The presented platform can be used to import such pre-normalized data for further analysis. Normalization of the mRNA microarray data were performed using the presented platform, including RMA background correction and quantile normalization.

The next step involved microarray significance analysis to calculate significant differences in the transcript levels. Statistically significant differences in the miRNA levels were found for only two pairs of cell groups, NHDF PK15 (P) versus NHDF (K) giving hsa-let-7e, hsa-miR-199a-5p, hsa-miR-99a and NHDF LPS PK15 (LP) versus NHDF LPS (L) giving hsa-miR-3197. The resulting list of statistically significant miRNA subsets was selected using a Median of False Discovery Rate (FDR) threshold of 0 (the threshold used is relative, which means that genes are significantly different at a level greater than 0.05) for the prediction of putative miRNA targets among the differentially expressed mRNAs.

**Fig. 12:**
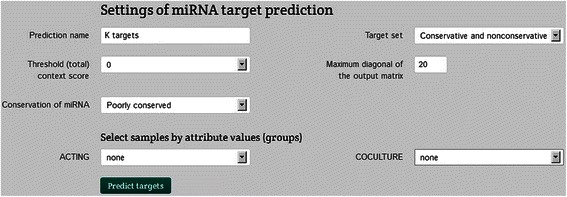
Selection panel for miRNA target prediction based on a single expression sample

**Fig. 13:**
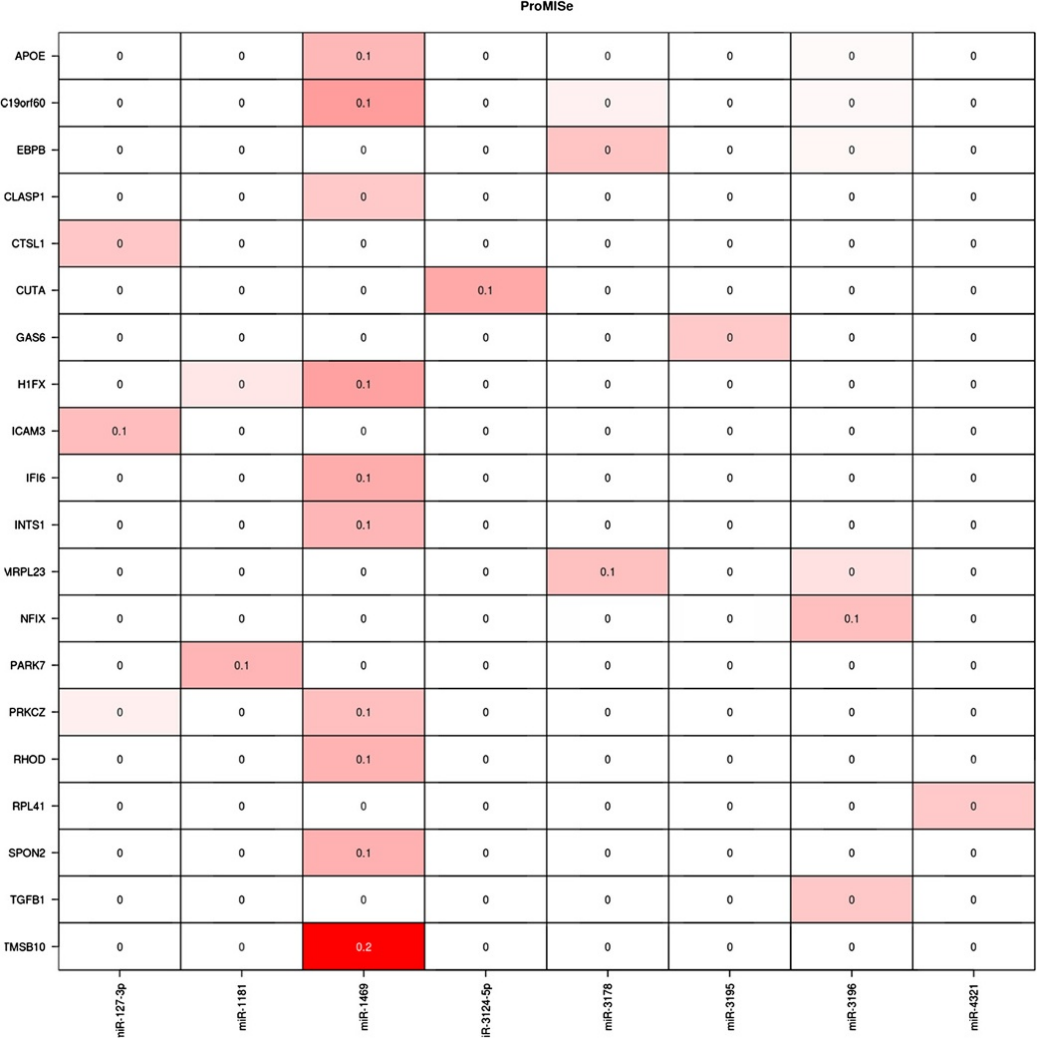
Section of the output table listing the ProMISe for the NHDF expression levels

The Integromics platform supports two miRNA target prediction methods. The first is based on a single expression sample (group). Due to the low diversity of the compared groups we selected the most liberal settings for the conservation of miRNA and target sites (Fig. 12). However, the results were found to be insignificant (Fig. 13).

The available parameters of this procedure include:

- the number of cells representing the highest probability. This parameter affects the dimension of the output matrix; e.g., a value of 10 results in an output matrix with diagonal length 10;
- the granularity level of the probe set (either transcript or gene);
- VB-GMM: the threshold that defines termination/convergence of the VB expectation maximization (VB-EM) algorithm, which is used to optimize the parameters;
- the maximum number of EM iterations of VB-GMM.

The ProMISe output tables for NHDF PK15 (P), NHDF LPS (L) and NHDF LPS PK15 (LP) were very similar to the NHDF results (see Fig. 13). The maximum probability (approximately 0.2) was obtained for the same pair (TMSB10 and hsa-miR-1469) from each sample.

The final component of the workflow used TargetScore for miRNA target prediction based on the variability of the expression of the compared groups. Using the TargetScore, our system prepares sequence-based information, the context score and PCT for each miRNA in the selected list. The exact approach depends on the selected level of analysis at either the gene or transcript level. The system informs the user whether sequence-based information was obtained for a given miRNA sample.

**Fig. 14:**
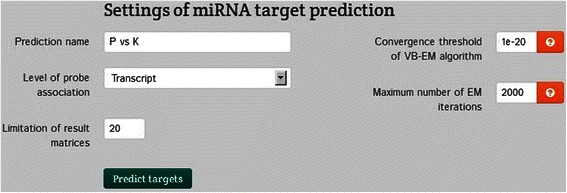
Selection panel for miRNA target prediction based on expression variability

The applied settings and parameters are presented above. We selected the transcript analysis level and the default values for the remaining settings (convergence threshold of the VB-EM algorithm and maximum number of EM iterations). The peak size of the resulting matrix was set at 20 (Fig. 14).

As a result of the above steps, we expected to answer the following questions: 1) which transcript (gene) displays the highest probability of being a target of any miRNAs; 2) which miRNA displays the highest probability of interfering with any transcripts; and 3) which miRNA-transcript (gene) pair displays the highest probability of interacting with each other?

The resulting tables present the peak probability values for the genes/transcripts. The attached histograms help distinguish this limited “peak” dataset from the entire population of results. If the “transcript” option is selected, the table also contains other transcripts associated with a specific gene according to the described multi-transcript protocol.

**Fig. 15:**
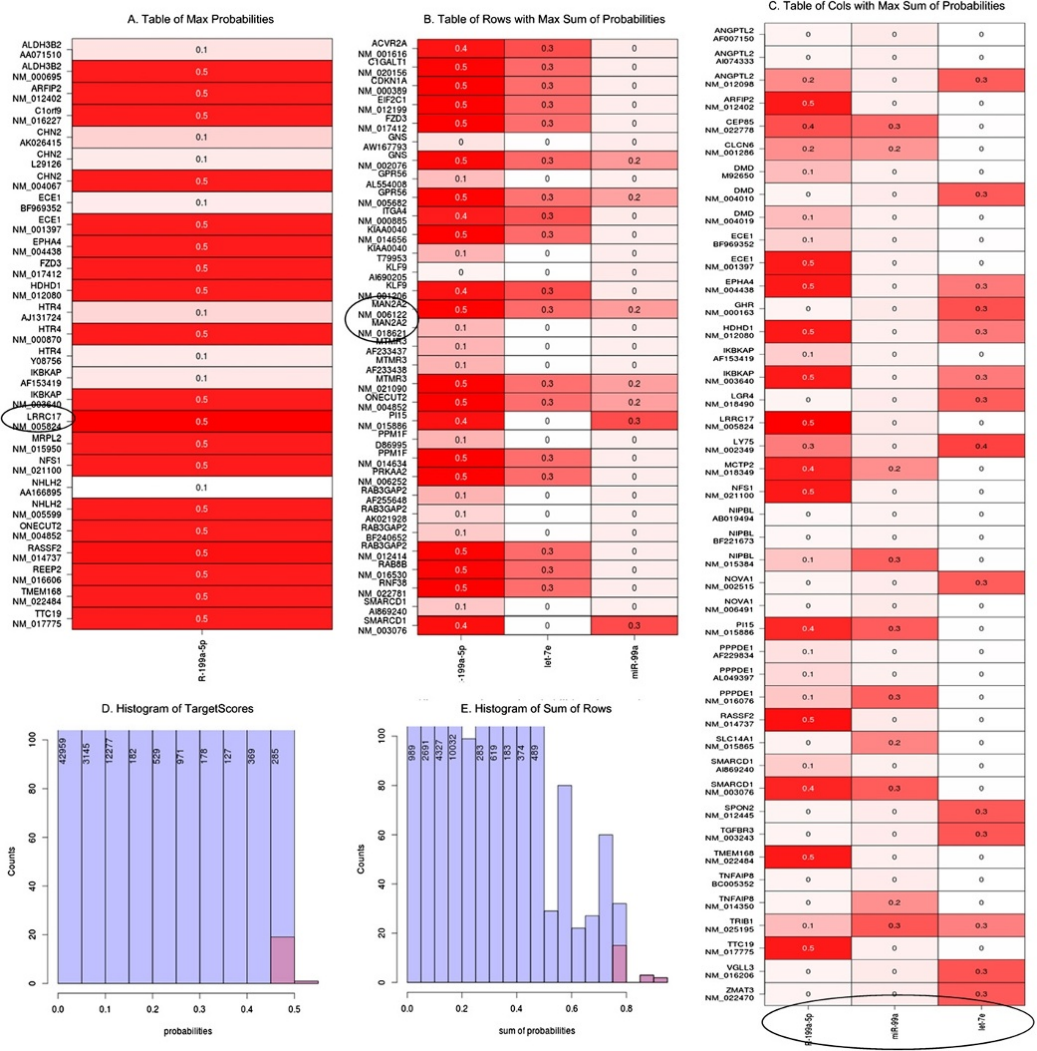
The most important part of result of TargetScore analysis NHDF PK15 (P) compared to NHDF (K). (**a**) The Table of Max Probabilities showing cells displaying the greatest probability of miRNA-mRNA interaction with NM_5824 (LRRC17 gene) marked. This transcript exhibits the highest probability from among mRNA/miRNA pairs (**b**) Table of rows with Max Sum of Probabilities containing rows (genes/transcripts) with the maximum values of the sum of probabilities, with NM_006122 (MAN2A2 gene) and NM_18621 (the same MAN2A2 gene) transcripts highlighted. (**c**) Table of columns with Max Sum of Probabilities containing columns (miRNAs) with the maximum values of the sums of probabilities (arranged in the order of decreasing probability: hsa-miR-199a-5p, hsa-miR-99a, hsa-let7e). (**d**) Histogram of TargetScores containing the TargetScore-computed probabilities of miRNA-mRNA interaction. (**e**) Histogram of Sum of Rows containing the sums of the TargetScore probabilities for each row (gene/transcript)
- histogram of the sums of the TargetScore probabilities for each row (gene/transcript)
- table of columns (miRNAs) containing the maximum values of the sums of probabilities

The full list of output files and miRNA target prediction charts based on the variability of expression includes TargetScore probabilities calculated only for log(FC) input:

- list of miRNA samples that contain no sequence-based information (context score or PCT) in the TargetScan database
- list of transcripts that are linked to more than one gene
- vector of the peak-probability genes computed only using log(FC)
- histogram of the TargetScore probabilities for the fold-change in expression and TargetScore probabilities calculated using the sequence-based information:
- table of cell types displaying the greatest probabilities of miRNA-mRNA interaction
- histogram of the TargetScore-computed probabilities of miRNA-mRNA interaction
- table of rows (genes/transcripts) containing the maximum values of the sum of the probabilities (see Fig. 15)

The output files support the following interpretation. Transcript NM_5824 (LRRC17 gene) exhibits the highest probability among mRNA/miRNA pairs (this conclusion is based on the full TargetScore results and the Table of Max Probability; see Fig. 15). The distribution of probabilities shows that the highest value is not an outlier. This suggests that we could expect many miRNA/mRNA pairs with slightly lower probability values. We select the transcripts with the best miRNA interaction probabilities from among the full TargetScore results and from the table of rows listing maximum probability sums (see Fig. 15). The selected transcript, NM_006122, is encoded by the MAN2A2 gene. The corresponding histogram facilitates assessment of the specificity of each value. By analogy, we could assess miRNAs by using maximum probability sum columns to select the highest probability of interference with any transcripts. The resulting miRNAs, arranged in the order of decreasing probability, are: hsa-miR-199a-5p, hsa-miR-99a and hsa-let7e (see Fig. 15).

## Conclusions

The DNA Microarray Integromics Analysis Platform is a user-friendly, open-source online system that provides a panoply of computational tools for biological data analysis. Its web-based graphical user interface renders the platform more accessible to non-IT experts, enabling domain scientists to interact with it at each step of the microarray data analysis, and to integrate biological data from other sources.

Several web-based DNA microarray data analysis platforms currently exist. However, most of these platforms do not support the multi-omics approach as a means of enhancing insight into molecular biology mechanisms. Table 1 compares selected characteristics and features of the DNA Microarray Integromics Analysis Platform with those of other web-based tools.

**Table 1:** Comparison of the characteristics of popular microarray analysis tools

Platform characteristic	WebArrayDB [11]	ArrayMining [12]	Babelomics [13]	DNA Microarray integromics analysis platform
miRNA-mRNA target site recognition	-	-	-	+
Integration of transcriptomics and lipidomics	-	-	-	+
Analysis of genetic variation	-	-	+^3^	-
Functional profiling			+^1^	+^2^
Conditional analysis of gene expression based on ANN	-	-	-	+
Imports and exports data to EBI databases	-	-	-	+
Teamwork and collaboration	-	-	-	+
License and source code availability	GNU General Public License	Free for academic and non-commercial use; source code not available	Free; source code available	Free for academic and non-commercial use; source code available

^1^Includes text mining, knowledge- and network-based annotations and gene set enrichment analysis; ^2^includes knowledge- and network-based annotations and gene set enrichment analysis; ^3^includes association analysis and genotype stratification

Only the Babelomics [13] platform includes any algorithms for integrative analysis of transcriptomics, proteomics and genomics data. Integromics analyses for transcriptomics and lipidomics data or mRNA and miRNA data are available exclusively in the DNA Microarray Integromics Analysis Platform.

## Abbreviations

ANN: Artificial neural network
GUI: Graphical user interface
RMA: Robust multi-array average
GCRMA: Guanine cytosine robust multi-array analysis
CCA: Canonical correlation analysis
PLS: Partial least squares regression
